# Radial multi‐echo bSSFP and IDEAL chemical shift separation in k‐space for high‐speed 3D hyperpolarized ^13^C metabolic MRI

**DOI:** 10.1002/mrm.30614

**Published:** 2025-07-28

**Authors:** Zirun Wang, Martin Grashei, Johannes Fischer, Sandra Sühnel, Nadine Setzer, Marcel Awenius, Andreas Korzowski, Ali C. Özen, Maxim Zaitsev, Michael Bock, Franz Schilling, Andreas B. Schmidt, Christoph A. Müller

**Affiliations:** ^1^ Division of Medical Physics, Department of Radiology, Medical Center, Faculty of Medicine University of Freiburg Freiburg Germany; ^2^ Technical University of Munich, School of Medicine and Health, Department of Nuclear Medicine TUM University Hospital Munich Germany; ^3^ German Cancer Research Center (DKFZ) Heidelberg Germany; ^4^ Faculty of Physics and Astronomy University of Heidelberg Heidelberg Germany; ^5^ German Cancer Consortium (DKTK), partner site Munich and German Cancer Research Center (DKFZ) Heidelberg Germany; ^6^ Munich Institute of Biomedical Engineering Technical University of Munich Garching Germany; ^7^ German Cancer Consortium (DKTK), partner site Freiburg and German Cancer Research Center (DKFZ) Heidelberg Germany; ^8^ NVision Imaging Technologies GmbH Ulm Germany

**Keywords:** carbon‐13 hyperpolarization, IDEAL, metabolic MRI, radial MRI

## Abstract

**Purpose:**

Hyperpolarized (HP) carbon‐13 (^13^C) MRSI provides real‐time information about metabolic processes but lacks high temporal and spatial resolution. This study introduces a multi‐echo–balanced steady‐state free precession (ME‐bSSFP) method with a 3D radial readout trajectory in a spiral phyllotaxis pattern as a method for non‐Cartesian HP ^13^C MRSI that allows for flexible reconstruction with high spatiotemporal resolution.

**Methods:**

The approach uses ME‐bSSFP with iterative decomposition of echo asymmetry and least‐squares estimation (IDEAL) for separating ^13^C metabolite signals. The method was evaluated using thermally polarized ^13^C‐enriched phantoms and in vivo experiments in healthy rats injected with HP [1‐^13^C]pyruvate.

**Results:**

The method successfully acquired and separated thermally polarized and HP ^13^C metabolite signals, demonstrating its versatility and effectiveness. This approach allows for the extraction of high temporal‐ or high spatial‐resolution 3D MRI from the same measurement. In vivo, dynamic HP metabolite intensities were extracted at a temporal resolution of 16 ms, and dynamic 3D metabolite images were generated with an isotropic FOV of 356 mm and a spatial resolution of 5.56 mm in 4.8 s.

**Conclusion:**

The proposed radial ME‐bSSFP method with iterative decomposition of echo asymmetry and least‐squares estimation decomposition provides a flexible, efficient, and robust approach for HP ^13^C MRSI. It opens new possibilities for monitoring metabolic processes with unprecedented temporal resolution, generating dynamic 3D metabolite maps, and applying rapid acquisition and undersampling schemes. The method's ability to optimize imaging temporal resolution and facilitate kinetic modeling paves the way for innovative applications in dynamic HP metabolic MRI.

## 1 INTRODUCTION

Hyperpolarized (HP) carbon‐13 (^13^C) MRSI is a powerful tool for dynamic imaging of metabolic processes in vivo. In HP ^13^C MRSI, metabolic contrast agents are prepared in a polarizer and administered intravenously, enabling real‐time imaging of their metabolic conversion within the body. Notably, MRSI of the ^13^C label exchange between HP [1‐^13^C]pyruvate and [1‐^13^C]lactate provides valuable information for clinical diagnostics of cancer by visualizing the Warburg effect and other pathologies.^1^

An HP spin ensemble exists in a transient spin state featuring a non‐thermal–equilibrium population, which dramatically enhances MRSI sensitivity by several orders of magnitude.^2^ However, T_1_ relaxation, the application of RF pulses, and T_2_ relaxation constantly and irreversibly deplete the HP magnetization, effectively limiting HP ^13^C MRSI to small FOVs and low spatiotemporal resolutions. Therefore, specialized signal excitation and acquisition strategies are required to efficiently sample the HP magnetization and metabolic conversions at high resolutions.

For research, many fast HP ^13^C MRSI methods are currently available.^3^, ^4^ They can be divided into three categories: fast spectroscopic imaging, model‐based acquisition strategies, and metabolite‐specific imaging.^3^ All methods involve various advantages and disadvantages depending on the imaging experiment. For conciseness, the following reviews are recommended.^5^, ^6^ Multi‐echo balanced steady‐state free precession (ME‐bSSFP) is a model‐based acquisition strategy for HP MRI that uses a nonselective RF excitation sequence with a low flip angle, short TR, and 180° excitation phase increments^7^ in combination with a multi‐echo readout that encodes chemical shift using multiple TEs.^8^, ^9^, ^10^, ^11^, ^12^, ^13^ With bSSFP, the long T_1_ and T_2_ relaxation times of common metabolites and the use of low flip angles (typically <30°) enable efficient HP ^13^C signal usage.^14^ A method for separating ^13^C metabolite multi‐echo signals is iterative decomposition of echo asymmetry and least‐squares estimation (IDEAL), which uses prior knowledge of the chemical shifts of the metabolic products formed during HP MRI experiment. It can be applied in either image space^9^, ^10^ or k‐space.^15^, ^16^, ^17^ The latter is especially suitable for non‐Cartesian MRI, that is, golden‐angle radial MRI, which features motion robustness, benign undersampling artifacts, and sliding‐window reconstructions.^18^, ^19^, ^20^ It further allows for the correction of chemical shift displacement before image reconstruction.

This work demonstrates an ME‐bSSFP approach with a radial readout trajectory in a 3D spiral phyllotaxis pattern^21^ as a new acquisition method for non‐Cartesian HP ^13^C MRSI. The 3D radial ME‐bSSFP was tested in vitro in experiments with ^13^C‐labeled thermally polarized phantoms and in an in vivo experiment using healthy rats following an injection of HP [1‐^13^C]pyruvate. Both thermally polarized and HP metabolite signals were successfully acquired and separated, and advantages were demonstrated, such as flexibility, efficiency, and robustness for HP ^13^C MRI with large FOVs at high spatiotemporal resolution.

## 2 METHODS

### 2.1 ^13^ C‐labeled reference solutions

For RF frequency and transmit power adjustments, 5 mL glass vials (15 mm i.d., 40 mm height) containing highly concentrated ^13^C‐labeled aqueous solutions of [1‐^13^C]lactate (5 mL, 2.23 M) or [^13^C]urea (5 mL, 4.0 M) were used in the in vitro experiments. For the in vivo experiments, two test glasses (10 mm i.d., 110 mm length) filled with aqueous solutions of ^13^C‐labeled [1‐^13^C]lactate (5 mL, 2.23 M) and [^13^C]urea (6 mL, 4.5 M, 8 mM gadoteric acid) were placed next to the animals as a reference. All isotope‐labeled compounds, that is, sodium L‐lactate‐1‐^13^C (≥99 atom % ^13^C, CAS: 81273‐81‐6) and urea‐^13^C (research grade, 99 atom % ^13^C, CAS: 58069‐82‐2) were purchased from Cambridge Isotopes Laboratories (Tewksbury, MA) or Merck (Darmstadt, Germany), and the solutions were prepared in‐house.

### 2.2 Hyperpolarization

For hyperpolarization of [1‐^13^C]pyruvate, 50 mg of [1‐^13^C]pyruvic acid (Merck) was polarized in a mixture containing 16 mM OX063 (Oxford Instruments, Abingdon, UK) and 1 mM gadoteric acid (Gd‐DOTA, (Guerbet, Villepinte, France) using a HyperSense DNP Polarizer (Oxford Instruments) at 3.35 T and 1.2 K under microwave irradiation at 94.133 GHz and 100 mW until saturation polarization was reached. Dissolution was performed with a prebuffered, preheated deuterium oxide  solution containing 18 mM phosphate and 0.1 g/L ethylenediaminetetraacetic acid, resulting in a final pyruvate concentration of 80 mM in an aqueous solution with a physiological pH of 7.98 ± 0.46. Note that deuterium oxide was utilized for dissolution in this preclinical study due to its common application in animal experiments.

### 2.3 Animal handling

All animal experiments were approved by an ethical review board (Regierung von Oberbayern, ROB‐55.2‐2532.Vet_02‐17‐177 and ROB‐55.2‐2532.Vet_02‐23‐70). For the in vivo MRI experiments, a healthy female Wistar rat (weight 273 g, Charles River, 17 weeks old) and three healthy male Wistar rats (weight 483.3 ± 2 g, Charles River, 14 weeks old) were used. Before imaging, anesthesia was initiated using 5% isoflurane in 100% oxygen as carrier gas. After catheterizing the tail vein, the animal was positioned in the isocenter of the magnet. The animal's rectal temperature was monitored using an MR‐compatible temperature monitoring system, Model 1030 (SA Instruments, Stony Brook, NY, USA), and maintained at 38°C by blowing warm air through the magnet bore using a Mistral‐Air Plus (The37°Company, Amersfoort, Netherlands). The breathing rate was continuously monitored during the MRI experiments using an electrocardiogram trigger unit equipped with a breathing pad (SA Instruments). The isoflurane concentration of the anesthesia gas was manually adjusted around 2% to keep the breathing rate at 50–70 breaths/min. For in vivo HP ^13^C measurements, the rats were injected twice with a total dose of 7.5 mL/kg (female) or 10 mL/kg (male) body weight via the tail vein catheter. The injections started 28.4 ± 3.2 s after the start of dynamic nuclear polarization (DNP) dissolution and lasted *t*_inj_ = 16.3 ± 1.4 s. After completion of the imaging protocol, the rats were euthanized under deep isoflurane (5%) anesthesia by intravenous injection of 200–400 mg/kg pentobarbital.

### 2.4 ^13^C MRI

Thermally polarized in vitro MRI experiments with the ^13^C‐labeled reference solutions were conducted on a 3 T Prisma (Siemens Healthineers, Erlangen, Germany) equipped with a dual‐tuned ^1^H/^13^C‐transmit/receive volume coil (Rapid Biomedical, Rimpar, Germany). In vivo HP ^13^C MRI experiments were conducted on a 3 T Biograph mMR (Siemens Healthineers) with the same dual‐tuned volume coil for female rats, or with a dual‐tuned ^1^H/^13^C‐transmit/receive surface coil (Rapid Biomedical) for male rats. For ^1^H MRI, T_1_‐weighted MPRAGE and T_2_‐weighted turbo spin echo (TSE) protocols (Siemens Healthineers) were acquired for anatomical co‐registration with ^13^C MRI. B_0_ homogeneity was adjusted using the MRI product routine for advanced shimming via ^1^H field map acquisition, before ^13^C RF adjustments were conducted. ^13^C RF power calibration was performed manually by determining the reference voltage of a 1‐ms block pulse to reach a 90° flip angle in the [^13^C]urea reference solution. The MRI system's ^13^C central frequency was adjusted to the resonance peak of [^13^C]urea for the in vitro experiment, and to the [1‐^13^C]lactate resonance frequency for the in vivo experiments.

**FIGURE 1:**
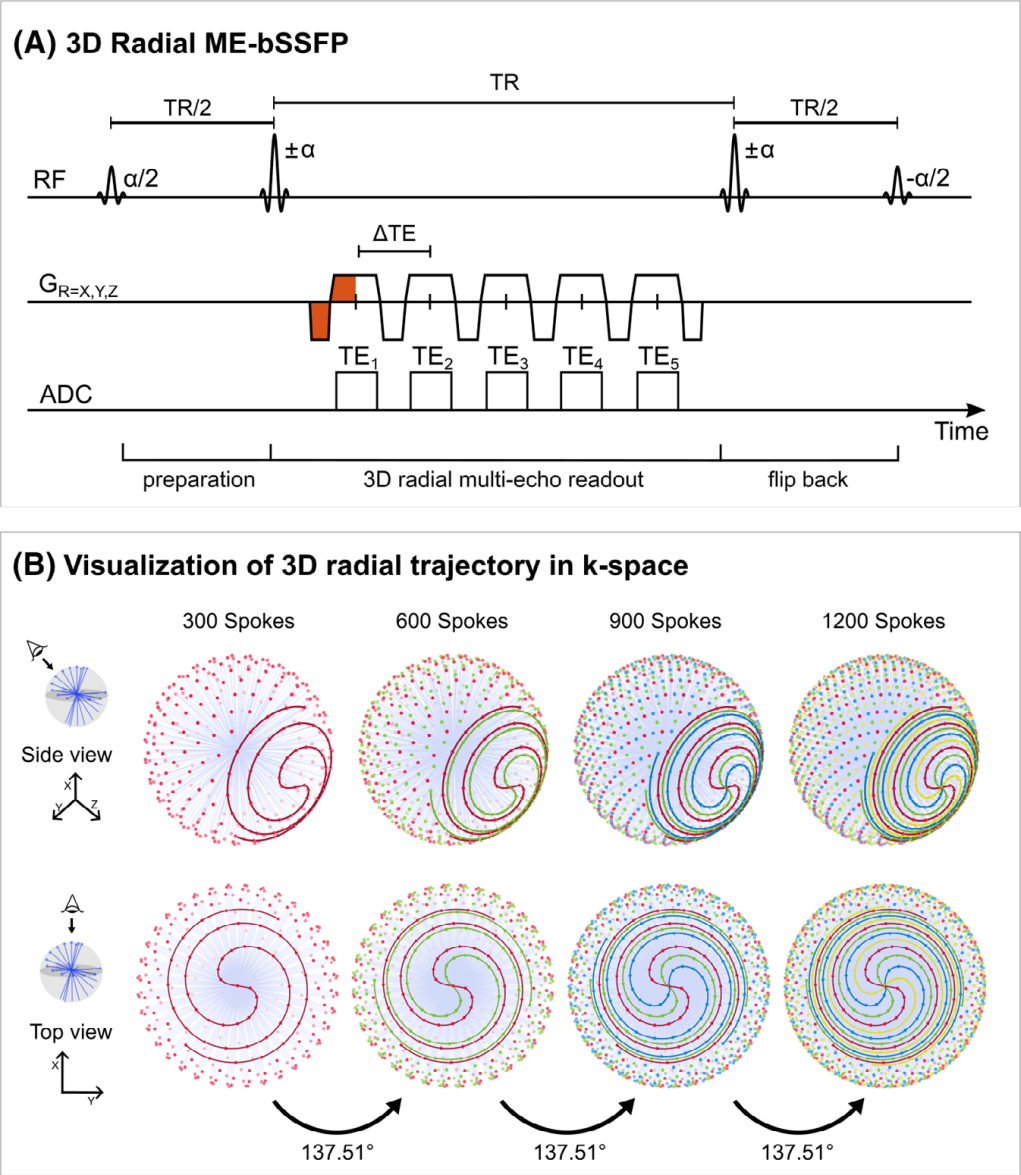
(A) Schematic diagram of 3D radial ME‐bSSFP sequence. Five echoes are acquired by monopolar trapezoidal gradients for IDEAL reconstruction, allowing the separation of signals from various ^13^C metabolites. All gradient moments are zero (balanced) in each echo (indicated by red area in G_R=X,Y,Z_) over the course of one TR. (B) Visualization of the spiral phyllotaxis sampling pattern in k‐space. Colored dots indicate the startpoint of the center‐through spokes. The four acquired measurements (300 spokes each) are differentiated by color. The colored spiral lines connecting the dots indicate the order of acquisitions for the spokes in each measurement. After the acquisition of each measurement, the sampling pattern is rotated by 137.51° around the z‐axis. ^13^C, carbon‐13; IDEAL, iterative decomposition of echo asymmetry and least‐squares estimation; ME‐bSSFP, multi‐echo‐balanced steady‐state free precession; G_R=X,Y,Z_, magnetic gradient along X, Y, Z axis.

For the ^13^C MRSI, the 3D ME‐bSSFP sequence (Figure 1A) was applied using the following parameters: alternating flip angles ±60° (in vitro) and ±10° (in vivo) after an initial *α*/2‐TR/2 preparation block; TR = 16 ms; five monopolar, center‐through gradient echoes with echo spacing of 2 ms, determined via an optimization process^22^ (see Figure S3), centered around TR/2 between excitation pulses. Readout bandwidth was 1000 Hz/px using a base resolution of 32 and an oversampling factor of 2. The FOV was set to 300 mm in vitro and 356 mm in vivo. Following the angular distributions as described by Piccini et al.,^21^ 300 radial orientations were acquired per measurement. After each measurement, the acquisition pattern was rotated by the golden angle (137.51°) about the z‐axis, following a fixed spiral phyllotaxis pattern (Figure 1B). The TR of 16 ms was optimized according to the chemical shifts of the main metabolites, pyruvate and lactate, to place both resonances in the in‐phase passbands of the bSSFP excitation profile (Figure S2). This resulted in an acquisition time of 4.8 s per measurement. In vivo, during the intravenous injection of HP [1‐^13^C]pyruvate, four dynamic radial ME‐bSSFP measurements were acquired before a magnetization tip‐back pulse (−*α*/2) to stabilize the magnetization. An interleaved ^13^C spectrum acquisition was conducted via a single‐shot nonlocalized free induction decay (FID) measurement with α = 10°, bandwidth = 6000 Hz, 4096 samples, and acquisition time = 1 s. This block of four radial ME‐bSSFP measurements and one FID was repeated three times.

### 2.5 Postprocessing

The entire postprocessing pipeline of ^13^C signal data was implemented in MatLab (R2022a, MathWorks, Natick, MA, USA). First, the raw signals of the nonlocalized FID acquisitions were reconstructed into spectra by applying an exponential filter corresponding to 5.9 Hz line broadening and Fourier transformation. The multi‐echo signals from the ME‐bSSFP measurements were decomposed via IDEAL using the relative ^13^C frequencies obtained from the spectra above: 0 Hz for [^13^C]urea and + 608 Hz for [1‐^13^C]lactate (in vitro); 15 Hz for [1‐^13^C]lactate, −190 Hz for [1‐^13^C]alanine, and − 370 Hz for [1‐^13^C]pyruvate (in vivo, e.g., female rat). This IDEAL metabolite decomposition was conducted in k‐space with chemical shift correction for full‐echo radial sampling, as described by Leupold et al.^13^ Notably, an iterative (*n* = 10 iteration steps) field inhomogeneity estimation was performed in k‐space by minimizing the residual between the estimated and corrected signal.

**FIGURE 2:**
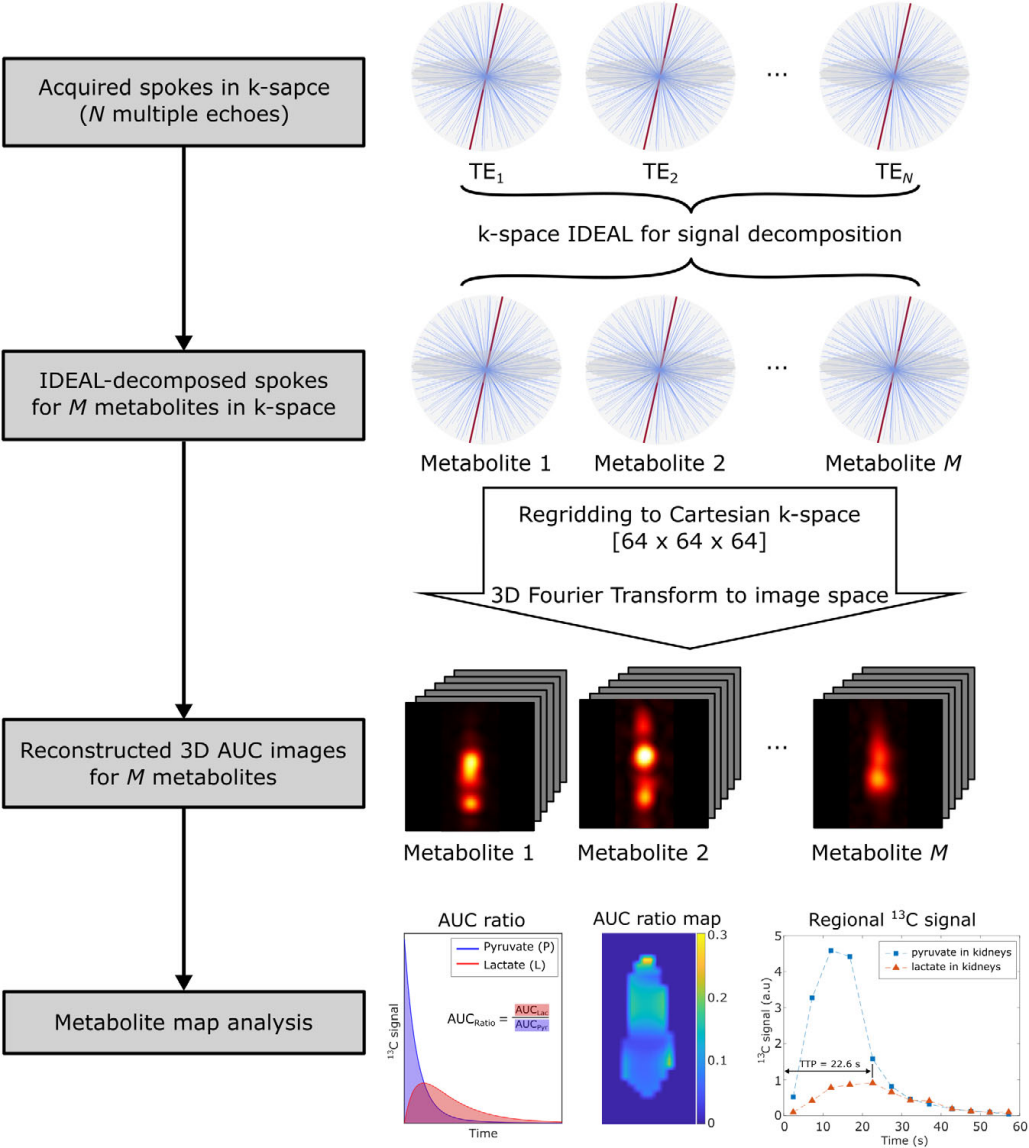
Visualization of the workflow of radial data processing and analysis. IDEAL decomposition algorithm was applied to each radial spoke (highlighted red line) in k‐space, followed by radial reconstruction, including density compensation, Kaiser‐Bessel regridding, and 3D fast Fourier transformation. AUC map calculation and regional time‐resolved dynamic signals were analyzed. AUC, area under the curve.

Image reconstruction was applied to the separated metabolite signals by an in‐house radial reconstruction pipeline, including density compensation, Kaiser‐Bessel regridding,^23^ fast Fourier transformation, and de‐apodization, transforming the radial k‐space data into a 3D image with a matrix size of 64 × 64 × 64 (Figure 2). Additionally, the dynamic k‐space data acquired over all radial ME‐bSSFP measurements were combined and reconstructed into one cumulative static 3D image per metabolite to generate spatially localized total metabolite signal maps.

In the reconstructed metabolite images, SNR was calculated, in the image domain, from the mean signal magnitude in a region of interest (ROI) and the noise magnitude outside the animal. The ROI was placed in the heart owing to its relatively high‐signal appearance. SNR values were corrected for the Rician distribution of magnitude noise signals.^24^ The lactate‐to‐pyruvate ratio (LP‐ratio) map was calculated via voxel‐wise division of lactate by pyruvate images. Background signals and non‐metabolism–related signals from the phantoms were excluded by manually applying a mask based on the pyruvate signal. For the in vivo data, a mean LP‐ratio value was quantified in the heart. The SDs of both the pyruvate and lactate signals in the heart ROI were propagated for error estimation of the LP‐ratio in the same ROI.

The global metabolite signal intensities were calculated by taking the mean of three signal samples around the origin of the separated metabolite k‐spaces. This data provided global dynamic metabolite signals with a high temporal resolution of 16 ms but no spatial localization. The global signal intensities of lactate and pyruvate were integrated over time to retrieve the area under the curve (AUC). The regional dynamic metabolite signals per ME‐bSFFP measurement (temporal resolution of 4.8 s) were extracted from the heart and kidney ROIs.

## 3 RESULTS

**FIGURE 3:**
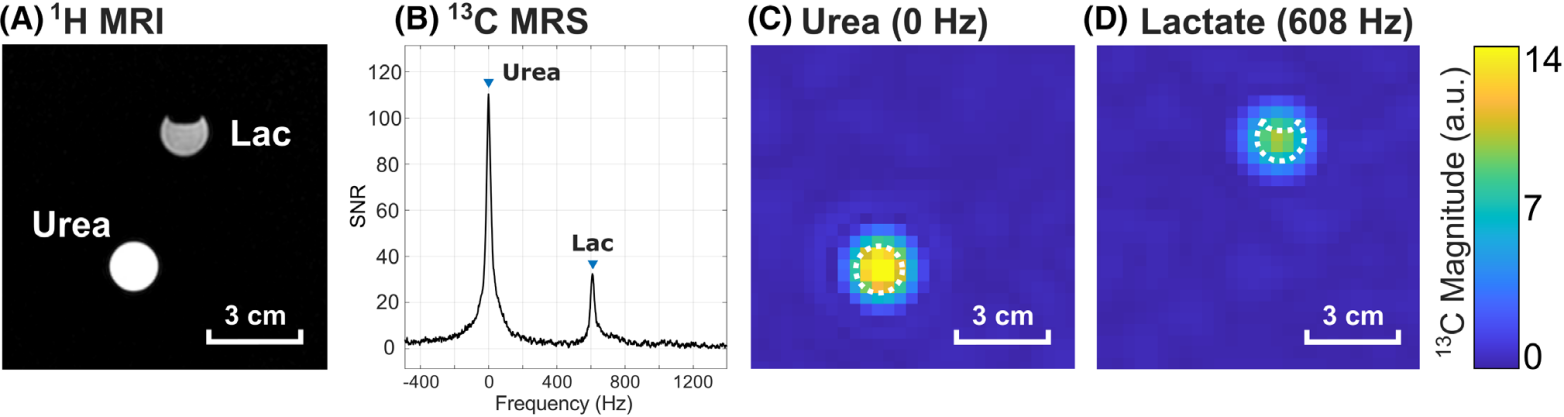
In vitro ^1^H MRI (A) of two thermally polarized aqueous ^13^C reference solutions. The MR spectrum (B) shows the ^13^C signals from 4.0 M [^13^C]urea and 2.23 M [1‐^13^C]lactate at 0 Hz and +608 Hz peak resonance frequency, respectively (TR = 10 s, six averages). The reconstructed ^13^C images show the urea ((C), SNR: 143) and lactate ((D), SNR: 85) signals decomposed via the IDEAL algorithm, with their locations well matching the reference ^1^H image (dashed white lines).^1^H, hydrogen.

Applying the IDEAL algorithm, the ^13^C signals from the thermally polarized in vitro phantoms (Figure 3A) measured with the radial ME‐bSSFP were successfully decomposed into those from urea and lactate at a frequency shift of 0 Hz and + 608 Hz, respectively. The frequencies for the decomposition were obtained from the nonlocalized ^13^C spectrum (Figure 3B). The reconstructed maps of the separated ^13^C metabolite signals correctly localized the two metabolite reference solutions without apparent signal leakage from one metabolite map to the other (Figure 3C,D).

**FIGURE 4:**
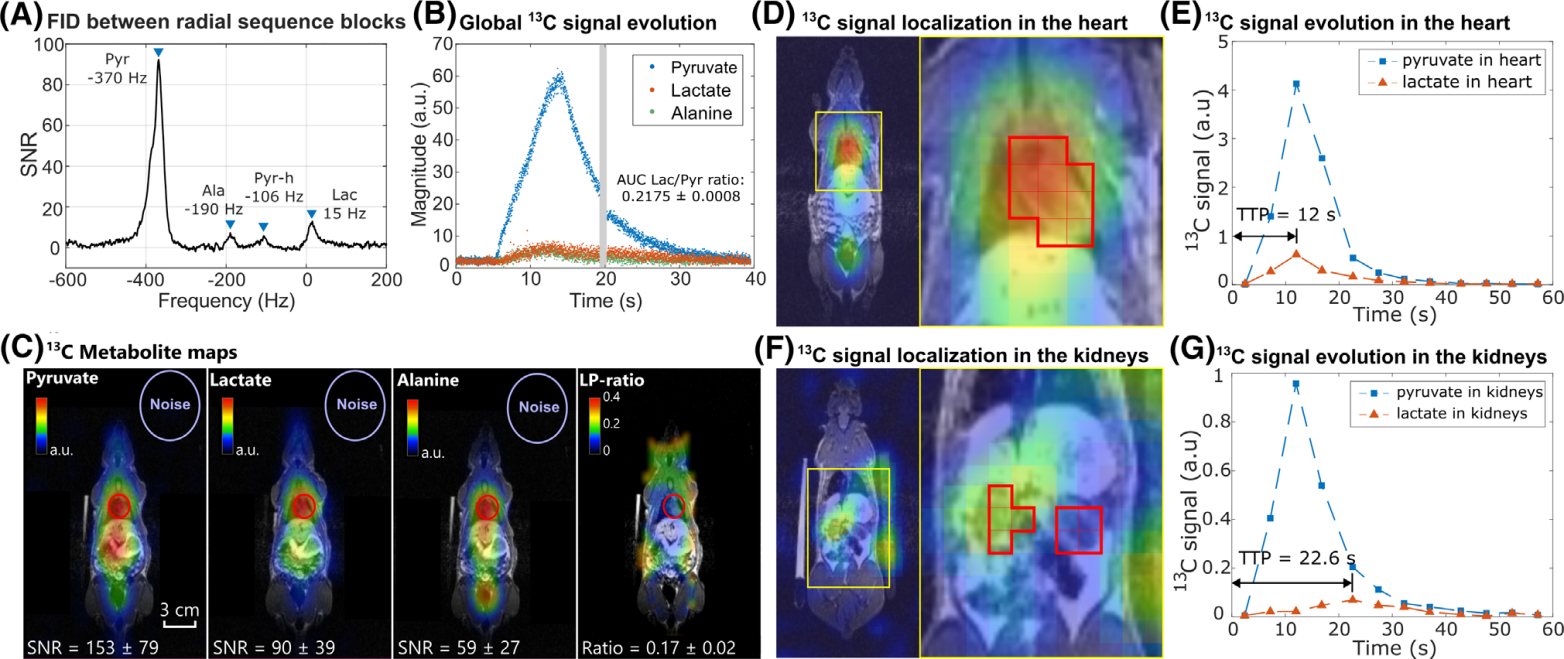
A female healthy rat imaged using a ^1^H/^13^C volume coil and 3 T MRI system. (A**)** In vivo ^13^C spectroscopy acquired 19.2 s after the sequence start, revealing the peak resonance frequencies of [1‐^13^C]pyruvate, [1‐^13^C]alanine, [1‐^13^C]pyruvate‐hydrate, and [1‐^13^C]lactate. (B) Dynamic, nonlocalized metabolite signal intensities obtained from the radial acquisition at the k‐space center after IDEAL signal decomposition, visualizing the pyruvate bolus injection and the subsequent conversion into lactate and alanine. Two nonlocalized spectroscopy scans (gray bar; the first shown in panel A) were interleaved between three blocks of ME‐bSSFP (the first two shown in panel B). (C) Central coronal slice of heart from HP ^13^C metabolite AUC images, obtained from radial ME‐bSSFP acquisition during the whole scan time (active metabolism from pyruvate to lactate and alanine). Voxel‐wise division of metabolite maps yielded the LP‐ratio map. Localized HP ^13^C signal evolutions from pyruvate and lactate extracted from the heart (D, E) and the kidneys (F, G) are presented, with the temporal resolution of 4.8 s (acquisition time for one measurement of 300 radial spokes). LP‐ratio, lactate‐to‐pyruvate ratio; TTP, time to peak.

In the in vivo experiment with the female rat, the peak resonance frequencies of the metabolites [1‐^13^C]pyruvate (−370 Hz, peak SNR: 92), [1‐^13^C]lactate (15 Hz, peak SNR: 13), [1‐^13^C]alanine (−190 Hz, peak SNR: 7.1), and [1‐^13^C]pyruvate‐hydrate (−106 Hz, peak SNR: 5.7) were obtained from the first nonlocalized FID acquisition after the HP pyruvate injection (Figure 4A). Using the spectral information as prior knowledge, the IDEAL decomposition of the multi‐echo signals successfully yielded metabolite‐separated k‐space signals for all ME‐bSSFP measurements. The metabolite‐separated k‐space signal intensities at center passage allowed the extraction of the global (nonlocalized) HP metabolite signal intensities with an effective 16 ms temporal resolution, depicting the arrival of the HP pyruvate bolus, the metabolic conversion of pyruvate to lactate and alanine, and the decay of all HP signals (Figure 4B).

Furthermore, the metabolite‐seperated k‐space signals from the ME‐bSSFP measurements were cumulated over all measurements and reconstructed into total metabolite maps with an isotropic spatial resolution of 5.56 mm, high SNR (mean pyruvate SNR = 153 ± 79 in heart ROI), and no temporal resolution (Figure 4C). Also, the signals were reconstructed individually for each measurement with the same spatial resolution and a temporal resolution of 4.8 s (Figure S4). From the latter, it was possible to extract the localized metabolite signal dynamics (Figure 4D–G), demonstrating a difference in the time‐to‐peak of dynamic lactate signal in the heart and kidney ROIs. As expected, strong ^13^C signals from pyruvate were detected in the heart, aorta, and vena cava along the central axis. Downstream lactate signals were also localized in the head, heart, and abdomen. The quantified signals in the heart ROI include contributions from both myocardial tissue and blood due to partial volume effects. Alanine resulted in the overall lowest SNR of the metabolite signals in both spectra and acquired metabolite maps.

**FIGURE 5:**
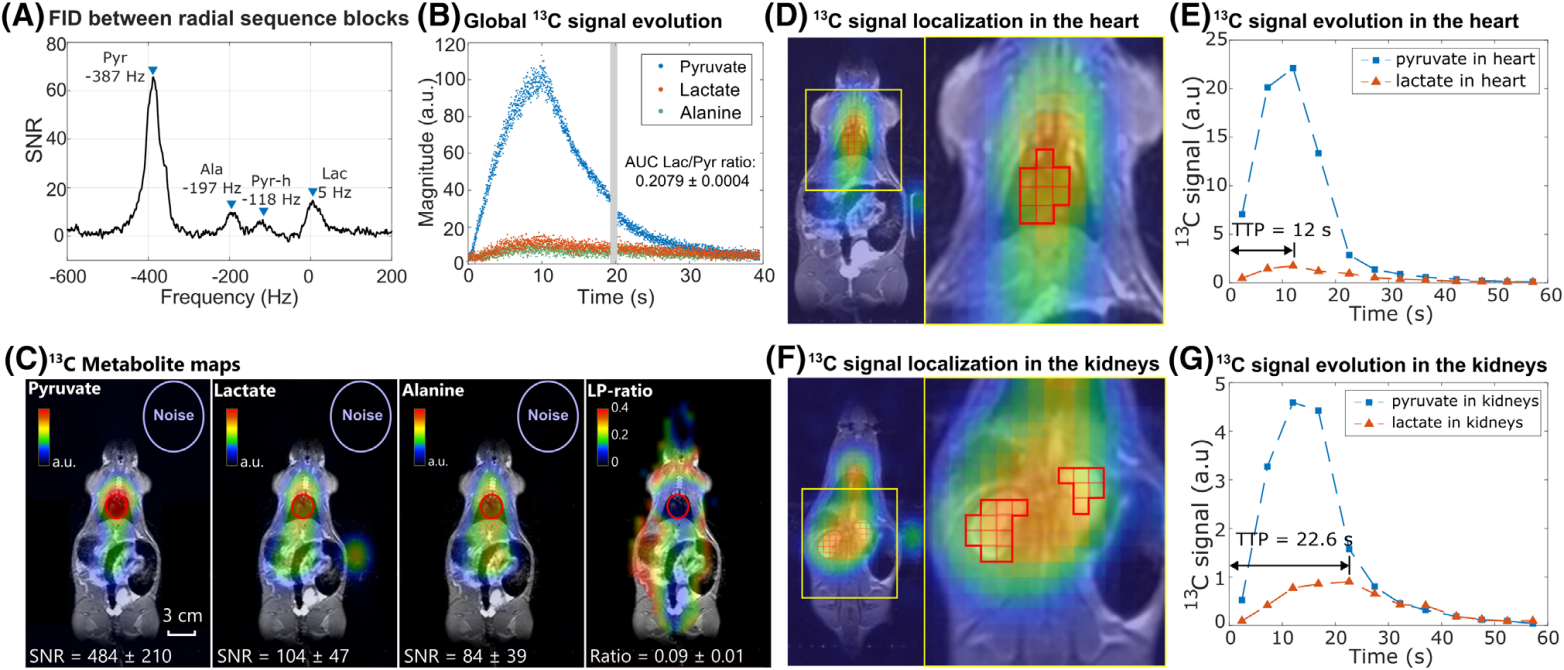
A male healthy rat imaged using a ^1^H/^13^C surface coil and 3 T MRI system. (A) In vivo ^13^C spectroscopy acquired 19.2 s after the sequence start, revealing the peak resonance frequencies of [1‐^13^C]pyruvate, [1‐^13^C]alanine, [1‐^13^C]pyruvate‐hydrate, and [1‐^13^C]lactate. (B) Dynamic, nonlocalized metabolite signal intensities obtained from the radial acquisition at the k‐space center after IDEAL signal decomposition, visualizing the pyruvate bolus injection and the subsequent conversion into lactate and alanine. Two nonlocalized spectroscopy scans (gray bar; the first shown in panel (A) were interleaved between three blocks of ME‐bSSFP (the first two shown in panel B). (C). Central coronal slice of heart from HP ^13^C metabolite AUC images, obtained from radial ME‐bSSFP acquisition during the whole scan time (active metabolism from pyruvate to lactate and alanine). Voxel‐wise division of metabolite maps yielded the LP‐ratio map. Localized HP ^13^C signal evolutions from pyruvate and lactate extracted from the heart (D, E) and the kidneys (F, G) are presented, with the temporal resolution of 4.8 s (acquisition time for one measurement of 300 radial spokes)

In a second in vivo experiment with a larger (484 g) male rat and using a surface coil with increased sensitivity, similar findings were successfully reproduced (Figure 5).

Renderings of the cumulative 3D ^13^C metabolite maps are provided as animated videos in Video S1. For completeness, the additional Supporting Information includes theoretical analyses of ME‐bSSFP signal behavior (Figures S1–S3), additional results from radial acquisitions in four rats (Figures S4–S7), and a comparative evaluation using Cartesian ME‐bSSFP (Figure S8) with IDEAL reconstruction, both in vitro (Figure S9) and in vivo (Figure S10).

## 4 DISCUSSION

The integration of ME‐bSSFP with center‐through radial trajectory and spiral phyllotaxis golden‐angle increments for MRI of HP ^13^C metabolites offers advantages over a Cartesian readout scheme. 3D multi‐echo radial bSSFP with IDEAL has already demonstrated its potential in ^1^H MRI applications.^25^, ^26^ Extending this method to HP ^13^C imaging presents unique benefits, as presented in the following. Radial echo‐planar spectroscopic imaging sequences, which record spectral information, have been investigated for HP ^13^C imaging.^27^, ^28^ In contrast to echo‐planar spectroscopic imaging, the multi‐echo readout with IDEAL separation allows fast acquisition of metabolites with high spatial resolution by using prior knowledge of the sparse ^13^C spectrum for reconstruction. Furthermore, the radial readout allows rapid detection of HP ^13^C metabolites in large FOVs and is known to be less sensitive to motion artifacts than Cartesian readout schemes.^18^ The combined methodologies are particularly advantageous in preclinical HP ^13^C MRSI settings involving whole‐animal FOVs, and would be suitable for clinical HP ^13^C MRSI of single organs or abdominal applications, such as free‐breathing MRI of the heart, kidneys, liver, or pancreas.^29^, ^30^, ^31^, ^32^

A direct comparison of our method to previous studies is challenging due to the many factors influencing acquisition parameters, and only a few studies have imaged rats on clinical MRIs. Awenius et al.^28^ achieved a 12^3^ matrix with 15 mm isotropic resolution every 6.3 s, whereas Tang et al.^33^ obtained 4 × 4 × 10 mm and 15 × 15 × 21 mm resolutions every 4s and 3.5 s for rat and human scans, respectively. Both studies employed dedicated rat transmit/receive coils. In contrast, our study used a larger volume coil designed for rabbits or a flexible surface coil, still achieving sufficient SNR in a 64^3^ 3D image matrix with 5.56 mm isotropic resolution and 4.8 s temporal resolution.

A flip angle of 10° was chosen based on the known theory of bSSFP signal evolution during the transient phase before a steady state is reached.^14^ The signal is dominantly T_1_‐weighted when a low flip angle and short TR (≪ T_2_) are set (see Figure S1), which has a positive effect on HP signal lifetime and the SNR in the dynamic measurements. bSSFP for MRSI can also be employed with ultralow (<1°) flip angle using a phase‐cycled frequency sweeping approach, as shown previously in a ^1^H MRSI study^34^ and HP ^13^C MRSI^35^, ^36^, ^37^ studies. Additionally, a nontypical 180° flip angle was used for HP ^13^C MR angiogram.^38^ A further reduction of the flip angle in the ME‐bSSFP measurements is therefore worth investigating.

To ensure optimal off‐resonant signal evolution during a bSSFP excitation pulse sequence, it is crucial to adjust the TR to match the expected metabolites' chemically shifted precession frequencies. Improper TR adjustments can lead to magnetization overlap with undesirable bands of the bSSFP frequency response profile, causing irreversible loss of HP magnetization, as occurred with the HP alanine signals in the experiments (Figure S2). Conversely, when the HP metabolites are located in the optimal bSSFP passbands, the stability of the HP signal during the acquisition window can be significantly improved. Small, highly concentrated ^13^C‐labeled reference solutions, for example, the [1‐^13^C]lactate solution, placed next to the subject, simplify the manual RF central frequency adjustment before the injection.

Extracting the metabolite signal intensities after the IDEAL decomposition from the k‐space center positions allows the metabolic conversion to be monitored at an extremely high sampling rate. Although this metabolic information is derived from nonlocalized data, the highly resolved dynamics may enhance biological kinetic modeling. In contrast, the temporal resolution of the presented localized dynamic MRSI is modest but sufficient to observe differences in time‐to‐peak of lactate production between organs. By incorporating all acquired radial readout lines into one cumulative metabolite image reconstruction, we optimize the SNR of the final metabolite and LP‐ratio maps. On the other hand, in MRI studies requiring high temporal and spatial resolution, for example, cardiac or vocal fold ^1^H MRI, radial k‐space sampling enables sliding temporal window reconstruction of subsets of the radial dataset.^18^, ^20^, ^39^, ^40^ Hence, applying sliding‐window reconstruction to HP ^13^C MRI, and utilizing the intrinsic sparsity of the groundtruth images, which is an observable trend in HP ^13^C MRI method development,^41^, ^42^, ^43^, ^44^ has high potential to yield highly resolved images in both temporal and spatial dimensions. Given the successful proof‐of‐concept using the radial ME‐bSSFP approach, this strategy warrants investigation as a next step.

Some limitations should be acknowledged. As a model‐based method, ME‐bSSFP relies on prior spectral knowledge, limiting the application for more complex spectra and new metabolite detection. Also, it comes at the cost of reduced experimental robustness if the model deviates from the real ^13^C spectrum, for example, under strong local B_0_ inhomogeneity. For future applications, acquiring a B_0_ map is recommended to correct the imaging data and improve IDEAL's field inhomogeneity estimation, especially if large FOVs are required. Furthermore, an accurate and robust method for frequency calibration, such as using the ^1^H water frequency as a reference for the ^13^C lactate one, might be considered to handle the B_0_ bias induced between the animal's interior and the exterior reference vial.

## 5 CONCLUSION

Radial ME‐bSSFP and generalized IDEAL chemical shift decomposition in k‐space feature high flexibility in data acquisition and reconstruction of hyperpolarized metabolites, opening up new possibilities: (a) monitoring nonlocalized metabolic conversion with an unprecedented temporal resolution; (b) extracting metabolite maps and ratio maps with high SNR and spatial resolution from the complete acquisition window. The successful demonstration of this method enables new perspectives, strategies, and applications in dynamic MRSI of hyperpolarized metabolites.

## FUNDING INFORMATION

Support was provided by the German Federal Ministry of Education and Research (BMBF) in the funding program Quantum Technologies–From Basic Research to Market Under the Project QuE‐MRT (contract number: 13N16448); the German Cancer Consortium (DKTK); the Research Commission of the University Medical Center Freiburg, B.E.S.T. Fluidsysteme GmbH I Swagelok Stuttgart; the German Research Foundation (DFG SCHM 3694/1‐1, SCHM 3694/2‐1, SFB1479, FI 2803/1‐1); and the Twinning of Magnetic Resonance Imaging Research Institutes (MRITwins, 101078393).

## CONFLICT OF INTEREST STATEMENT

c.a.m. is an employee of the company NVision Imaging Technologies GmbH. f.s. serves on the scientific advisory board of NVision Imaging Technologies GmbH.

## Supporting information




**Figure S1.** The calculated effective relaxation time (T_eff_) of bSSFP signal in the transient phase with fixed T_1_ = 30 s and T_2_ = 3 s based on the approximated equation from Scheffler et al. ^14^ When a low flip angle is used, T_eff_ is dominated by T_1_.
**Figure S2.** Numerical simulation results of bSSFP sequence across a broad bandwidth of off‐resonance frequencies (−500 Hz to 50 Hz) from solving the Bloch equations. Left: Periodic excitation profile of the bSSFP after 100, 300, and 600 refocusing pulses, showing stable passbands (orange: lactate 15 Hz, blue: pyruvate −370 Hz), unstable passbands (green: alanine −190 Hz), and stopbands (graygrey: −93 Hz). Right: Simulated signal evolution with time (number of pulses) of spin behaviors at different frequencies. Ideally, the metabolites‐of‐interest are placed into the stable passbands (e.g., lactate, pyruvate) to avoid artifacts induced by fast varied signal magnitude.
**Figure S3.** Simulation of the stability of bSSFP as function of echo spacing delta TE in multi‐echo readout. The inverse of matrix condition number (1/cond(A), upper row) reflects the sensitivity of the matrix inversion within IDEAL to any potential perturbation; 1 is optimal, and a low value would result in strong variations in the solution from only small perturbations in the measured signals. The effective number of signal average (NSA, middle and lower row) reflects the effective SNR level from the metabolites.^22^ ΔTE = 2 ms was chosen as the first optimal echo spacing considering the functionality of the ADC readout block. The calculations were based on 5 echoes of 2 frequencies (lactate: 0 Hz, pyruvate: −385 Hz) for NSA of echoes, and of 3 frequencies (lactate: 0 Hz, pyruvate: −385 Hz, alanine: −160 Hz) for NSA of different metabolites (mimicked). All echoes: both odd and even echoes are involved; odd/even echoes: only odd/even echoes are involved.
**Figure S4.** Results for rat F1
**Figure S5.** Results for rat M1
**Figure S6.** Results for rat M2
**Figure S7.** Results for rat M3
**Figure S8.** (A) Schematic representation of the dynamic 3D Cartesian ME‐bSSFP with bipolar multi‐echo readout in one direction and phase‐encoding in orthogonal and slice direction. (B) The post‐processing routine of the Cartesian ME‐bSSFP included echo image reconstruction and IDEAL decomposition in image space to obtain the individual metabolite maps.
**Figure S9.** Thermally polarized in vitro measurements with radial and Cartesian ME‐bSSFP were successfully reconstructed using IDEAL at the corresponding frequencies of 0 Hz (**C**, urea, SNR: 18) and + 608 Hz (**D**, lactate, SNR: 13), yielding the separated metabolite maps.
**Figure S10** (A) In vivo ^13^C spectroscopy at 25.6 s after the sequence start, revealing the peak resonance frequencies of [1−^13^C]pyruvate (220 Hz) and [1−^13^C]lactate (590 Hz). The thermal [^13^C]urea (0 Hz) peak is fixed at 0 Hz as prior knowledge from the thermally polarized [^13^C]urea solution. (B) Global signal level with time in every decomposed image. The Cartesian sampling provides a temporal resolution of 2.56 s, roughly depicting the injection bolus and conversion of pyruvate into lactate. (C) Central coronal slice of heart from HP ^13^C metabolite AUC images, obtained from Cartesian ME‐bSSFP acquisition after injection of HP pyruvate into the rat F2, showing metabolic conversion from pyruvate to lactate. A slice at the position of the reference solution highlights the thermally polarized [^13^C]urea next to the animal. Voxel‐wise division of metabolite maps and applying a mask based on the pyruvate signal yielded the lactate‐to‐pyruvate ratio map.

**Video S1.** Folder “*Radial 3D 13C Images*”: cumulative 3D metabolite images (pyruvate, lactate, alanine) and 3D lactate‐to‐pyruvate, alanine‐to‐pyruvate metabolite ratio map from radial ME‐bSSFP acquisitions of four rats (F1, M1, M2, M3).

**Video S2.** Folder “*Cartesian 3D 13C Images*”: cumulative 3D metabolite images (pyruvate, lactate) and 3D lactate‐to‐pyruvate metabolite ratio map from Cartesian ME‐bSSFP acquisition of rat F2.
