# Supplementary material for: Radial multi‐echo bSSFP and IDEAL chemical shift separation in k‐space for high‐speed 3D hyperpolarized 13C metabolic MRI
Source: Magn Reson Med. 2025 Jul 28;94(5):2190–200. doi: 10.1002/mrm.30614 (PMC12393183; doi:10.1002/mrm.30614)
Supplement: Supplementary file 1 — Figure S1. The calculated effective relaxation time (Teff) of bSSFP signal in the transient phase with fixed T1 = 30 s and T2 = 3 s based on the approximated equation from Scheffler et al. 14 When a low flip angle is used, Teff is dominated by T1. Figure S2. Numerical simulation results of bSSFP sequence across a broad bandwidth of off‐resonance frequencies (−500 Hz to 50 Hz) from solving the Bloch equations. Left: Periodic excitation profile of the bSSFP after 100, 300, and 600 refocusing pulses, showing stable passbands (orange: lactate 15 Hz, blue: pyruvate −370 Hz), unstable passbands (green: alanine −190 Hz), and stopbands (graygrey: −93 Hz). Right: Simulated signal evolution with time (number of pulses) of spin behaviors at different frequencies. Ideally, the metabolites‐of‐interest are placed into the stable passbands (e.g., lactate, pyruvate) to avoid artifacts induced by fast varied signal magnitude. Figure S3. Simulation of the stability of bSSFP as function of echo spacing delta TE in multi‐echo readout. The inverse of matrix condition number (1/cond(A), upper row) reflects the sensitivity of the matrix inversion within IDEAL to any potential perturbation; 1 is optimal, and a low value would result in strong variations in the solution from only small perturbations in the measured signals. The effective number of signal average (NSA, middle and lower row) reflects the effective SNR level from the metabolites.22 ΔTE = 2 ms was chosen as the first optimal echo spacing considering the functionality of the ADC readout block. The calculations were based on 5 echoes of 2 frequencies (lactate: 0 Hz, pyruvate: −385 Hz) for NSA of echoes, and of 3 frequencies (lactate: 0 Hz, pyruvate: −385 Hz, alanine: −160 Hz) for NSA of different metabolites (mimicked). All echoes: both odd and even echoes are involved; odd/even echoes: only odd/even echoes are involved. Figure S4. Results for rat F1 Figure S5. Results for rat M1 Figure S6. Results for rat M2 Figure S7. Results f [file MRM-94-2190-s003.docx]

**Radial multi-echo bSSFP and IDEAL chemical shift separation in k-space for high-speed 3D hyperpolarized ^13^C metabolic MRI**

**Authors:**

Zirun Wang^1^, Martin Grashei^2^, Johannes Fischer^1^, Sandra Sühnel^2^, Nadine Setzer^2^, Marcel Awenius^3,4^, Andreas Korzowski^3^, Ali C. Özen^1^, Maxim Zaitsev^1^, Michael Bock^1^, Franz Schilling^2,5,6^, Andreas B. Schmidt^1,7,#,^*, and Christoph A. Müller^1,7,8,#^

1. Division of Medical Physics, Department of Radiology, Medical Center, Faculty of Medicine, University of Freiburg, Germany

2. Technical University of Munich, School of Medicine and Health, Department of Nuclear Medicine, TUM University Hospital, Munich, Germany

3. German Cancer Research Center (DKFZ), Germany

4. Faculty of Physics and Astronomy, University of Heidelberg, Heidelberg, Germany

5. German Cancer Consortium (DKTK), partner site Munich and German Cancer Research Center (DKFZ), Germany

6. Munich Institute of Biomedical Engineering, Technical University of Munich, Garching, Germany

7. German Cancer Consortium (DKTK), partner site Freiburg and German Cancer Research Center (DKFZ), Germany

8. NVision Imaging Technologies GmbH, Ulm, Germany

# contributed equally

* [andreas.schmidt@uniklinik-freiburg.de](mailto:andreas.schmidt@uniklinik-freiburg.de)

#

# **Supplementary Information (SI):**

### The Supplementary Information (SI) provides additional theoretical background and experimental data that support the main body of this paper. It is structured as follows:

### **Theoretical Section**

### This part includes simulation-based analyses to provide physical insights into the signal behavior of the ME-bSSFP sequence:

### Analytical search of the proper flip angle based on the effective HP signal decay rate under the alternative excitation

### Bloch simulations of the signal evolution from on/off-resonance frequencies

### Echo spacing optimization based on the calculations of matrix condition number and effective number of signal average

### **Additional Results from 3D Radial ME-bSSFP Scans**

### This section presents AUC and dynamic metabolic imaging data acquired from four rats (F1, M1, M2, and M3) using the 3D radial ME-bSSFP sequence.

### **3D Cartesian ME-bSSFP with IDEAL-Based Post-Processing**

### To provide a basis for comparison, this section includes a parallel evaluation using Cartesian ME-bSSFP and image-domain IDEAL reconstruction:

- Cartesian sequence diagram and post-processing pipeline
- *In vitro* experiments: comparison of radial vs. Cartesian encoding
- *In vivo* results acquired from Rat F2, demonstrating image quality and metabolite separation under Cartesian encoding

### **1. Numerical simulations**

Based on the approximated equation from Scheffler et al. ^14^, an analytical search of the proper flip angle for the refocusing RF pulse excitation was performed. A low flip angle of 10° was applied so that the decay of the transient HP signal under bSSFP is almost entirely T_1_-weighted (**Figure S1**).


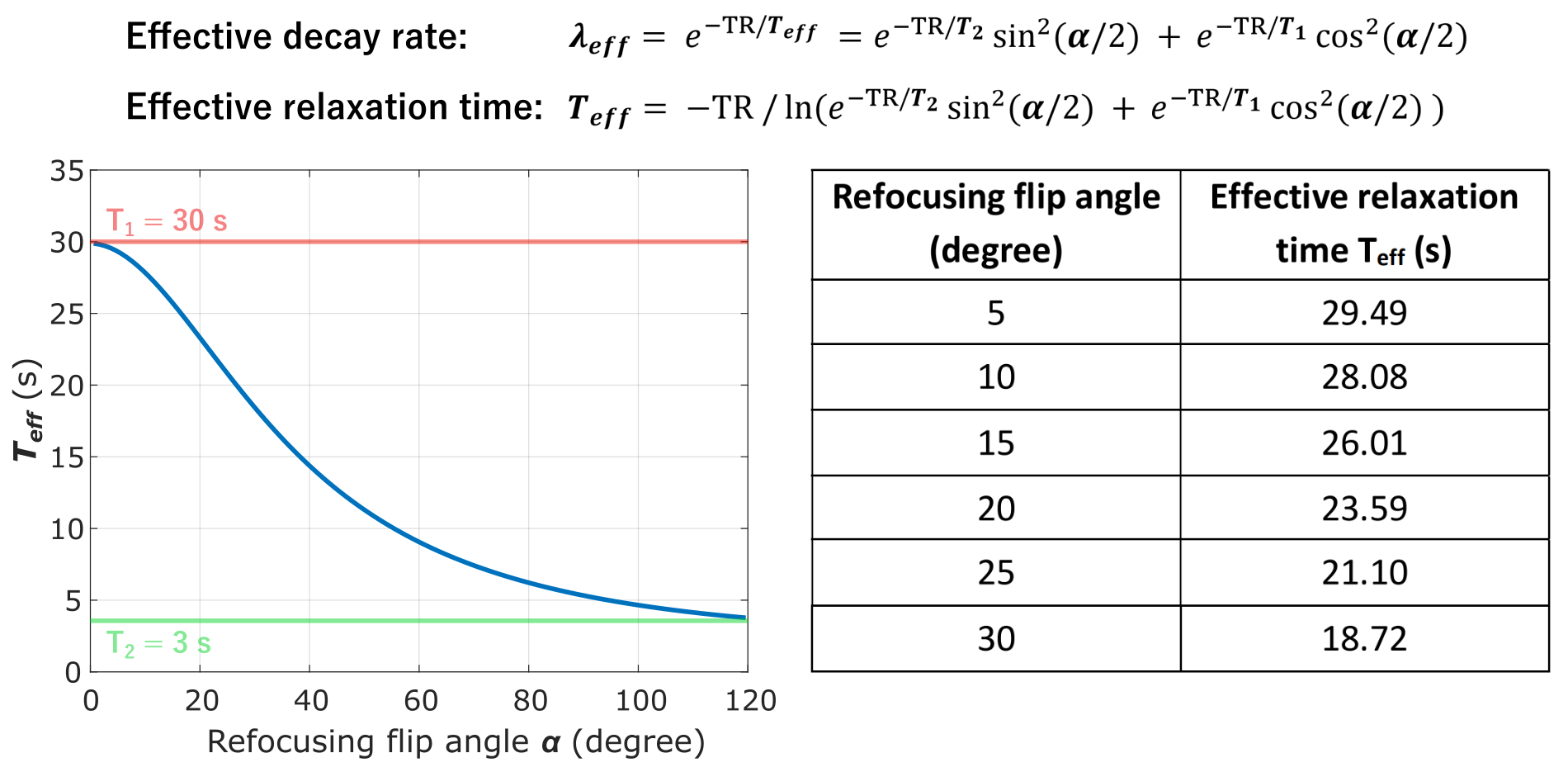


**Figure** **S1.** The calculated effective relaxation time (T_eff_) of bSSFP signal in the transient phase with fixed T_1_ = 30 s and T_2_ = 3 s based on the approximated equation from Scheffler et al. ^14^. When a low flip angle is used, T_eff_ is dominated by T_1_.

The bSSFP frequency sensitivity profiles were simulated using isochromats ranging from -500 Hz to 50 Hz. The simulations were based on Bloch equations, mimicking the *in vivo* excitation (TR = 16 ms, α = ±10°, Magnetization_start_ = 10000, Magnetization_equilibrium_ = 1, *α*/2-TR/2 preparation included). We showed the simulated profiles at three time points (100, 300, 600 pulses applied) and the spin signal evolutions in different response bands during the simulated pulse sequence (**Figure** **S2**). Our result is consistent with the relevant study ^14^, which supports using flip angles below 30° for transient-phase signals.


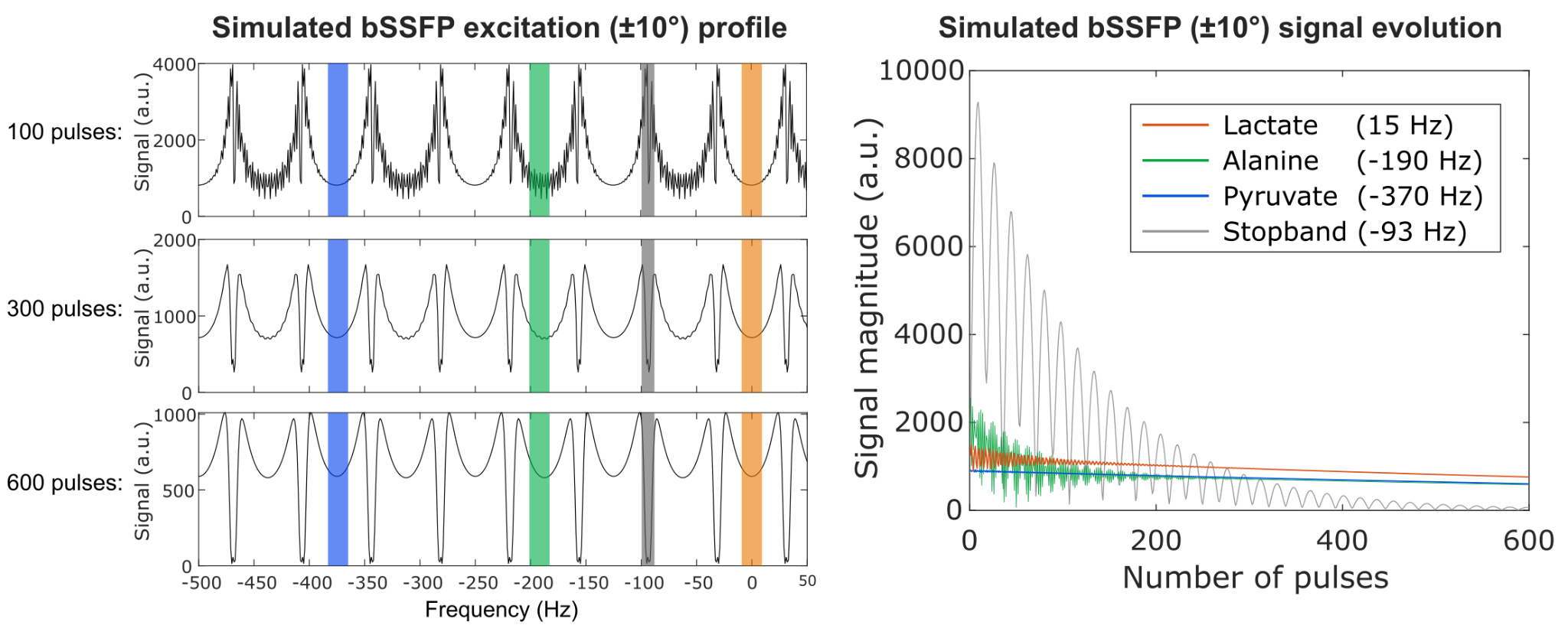


**Figure** **S2.** Numerical simulation results of bSSFP sequence across a broad bandwidth of off-resonance frequencies (-500 Hz to 50 Hz) from solving the Bloch equations. **Left:** Periodic excitation profile of the bSSFP after 100, 300, and 600 refocusing pulses, showing stable passbands (orange: lactate 15 Hz, blue: pyruvate -370 Hz), unstable passbands (green: alanine -190 Hz), and stopbands (grey: -93 Hz). **Right:** Simulated signal evolution with time (number of pulses) of spin behaviors at different frequencies. Ideally, the metabolites-of-interest are placed into the stable passbands (e.g., lactate, pyruvate) to avoid artifacts induced by fast varied signal magnitude.

The echo spacing was determined via an optimization process. We simulated the impact of different echo spacing choices on the effective signals, optimizing the Matrix Condition Number and the Effective Number of Signal Averages (NSA), shown in **Figure S3**, as described by Reeder SB et al. ^22^. ΔTE = 2 ms was chosen as the first realizable echo spacing (red band) considering the functionality of the ADC readout block, yielding in simulations a matrix condition number of 0.9 (1 is ideal), effective NSA 4.9 for echoes and NSA 4.6 for metabolites out of 5 acquired echoes.


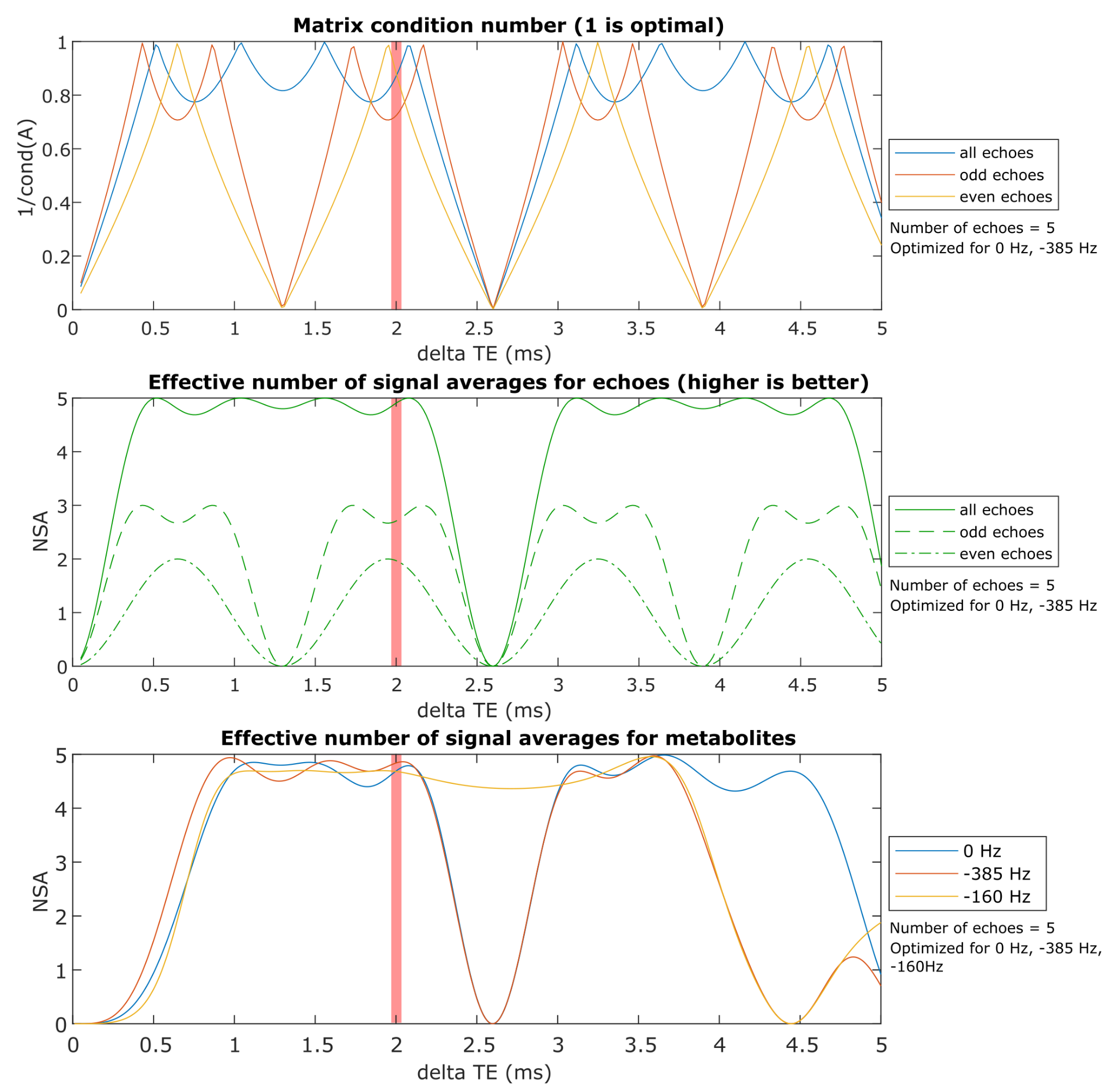


**Figure** **S3.** Simulation of the stability of bSSFP as function of echo spacing delta TE in multi-echo readout. The inverse of matrix condition number (1/cond(A), **upper row**) reflects the sensitivity of the matrix inversion within IDEAL to any potential perturbation; 1 is optimal, and a low value would result in strong variations in the solution from only small perturbations in the measured signals. The effective number of signal average (NSA, **middle** and **lower row**) reflects the effective SNR level from the metabolites ^22^. ΔTE = 2 ms was chosen as the first realizable echo spacing (red band) considering the functionality of the ADC readout block. The calculations were based on 5 echoes of 2 frequencies (lactate: 0 Hz, pyruvate: -385 Hz) for NSA of echoes, and of 3 frequencies (lactate: 0 Hz, pyruvate: -385 Hz, alanine: -160 Hz) for NSA of different metabolites (mimicked). All echoes: both odd and even echoes are involved; odd/even echoes: only odd/even echoes are involved.

### **2. Additional Results from 3D Radial ME-bSSFP**

### In total, the proposed 3D radial ME-bSSFP combined with IDEAL decomposition method was tested on four healthy rats. One female rat (F1) was scanned by the ^1^H/^13^C dual-tuned surface coil (Rapid Biomedical): weight 273 g, Charles River, age 17 weeks old. Three male rats (M1, M2, M3) were scanned by the ^1^H/^13^C dual-tuned surface coil (Rapid Biomedical): weight 483.3 ± 2 g, Charles River, 14 weeks old. Identical animal handling, parameters for ^13^C MRI scan, and reconstruction pipeline as described in the main text were used. Due to the relatively large rodent size, the surface coil was centered on the abdomen for M1 and M2, and on the heart for M3, in order to detect HP signals from different parts of the animals.

The additional results are presented here, including: **A.** ^13^C spectrum acquired by non-localized FID spectroscopy; **B.** Non-localized ^13^C signal evolution of metabolites calculated from the decomposed near-center radial kspace spokes; **C.** Central coronal slice of heart from HP ^13^C for three metabolites, as well as the voxel-wise calculated AUC ratio map for lactate-to-pyruvate (LP-ratio); Localized HP ^13^C signal evolutions from pyruvate and lactate extracted from the heart (**D**, **E**) and the organs-of-interest (**F,** **G**); Dynamic metabolite image series at the organs-of-interest every 4.8 s for pyruvate (**H**) and lactate (**I**). The results for rat F1 (**Figure S4**), M1 (**Figure S5**), M2 (**Figure S6**), and M3 (**Figure S7**) are shown. The full 3D slices of the ^13^C metabolite maps are displayed in **Video S1**.

**Figure S4.** Results for rat F1


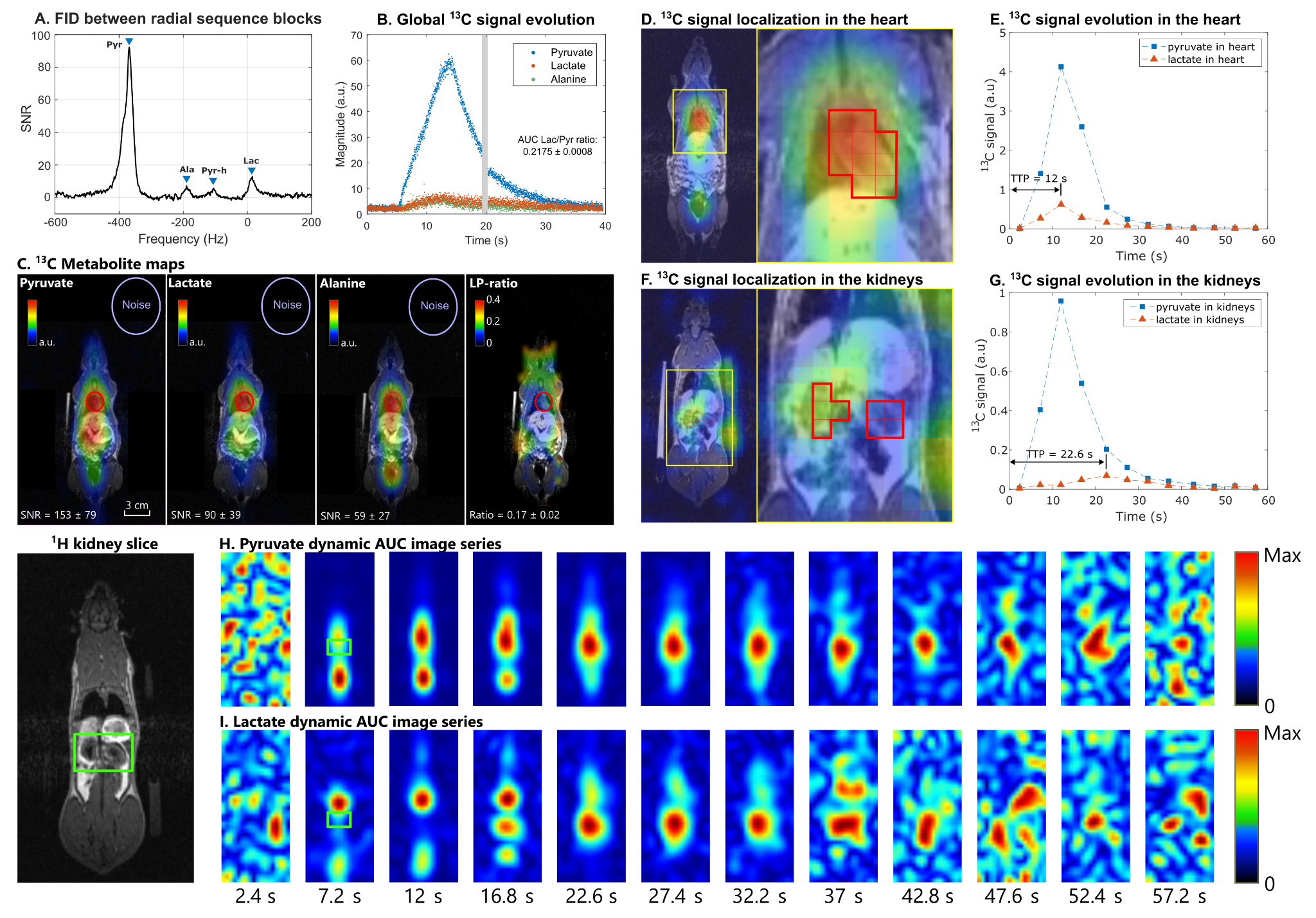


**Figure S5.** Results for rat M1


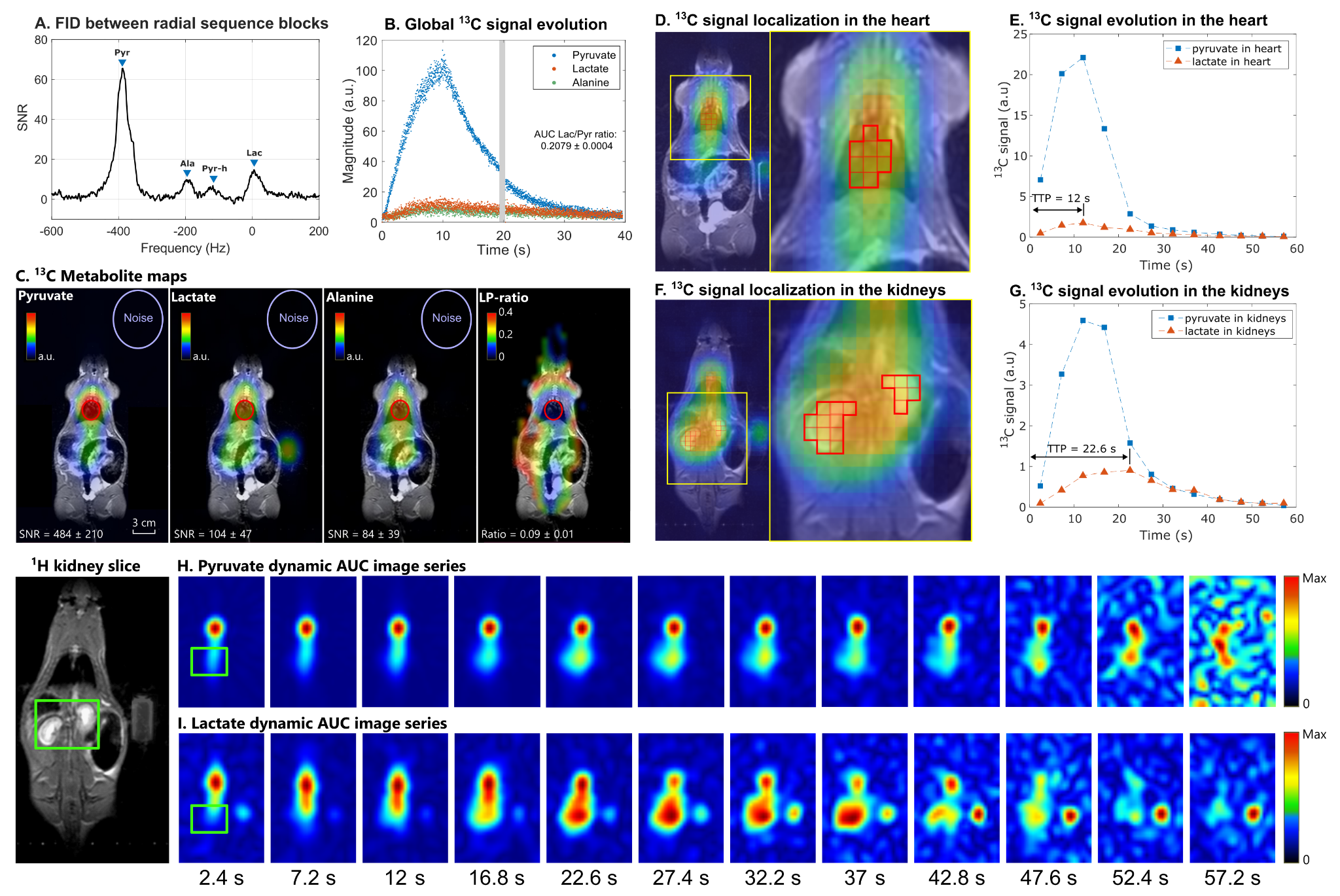


**Figure S6.** Results for rat M2


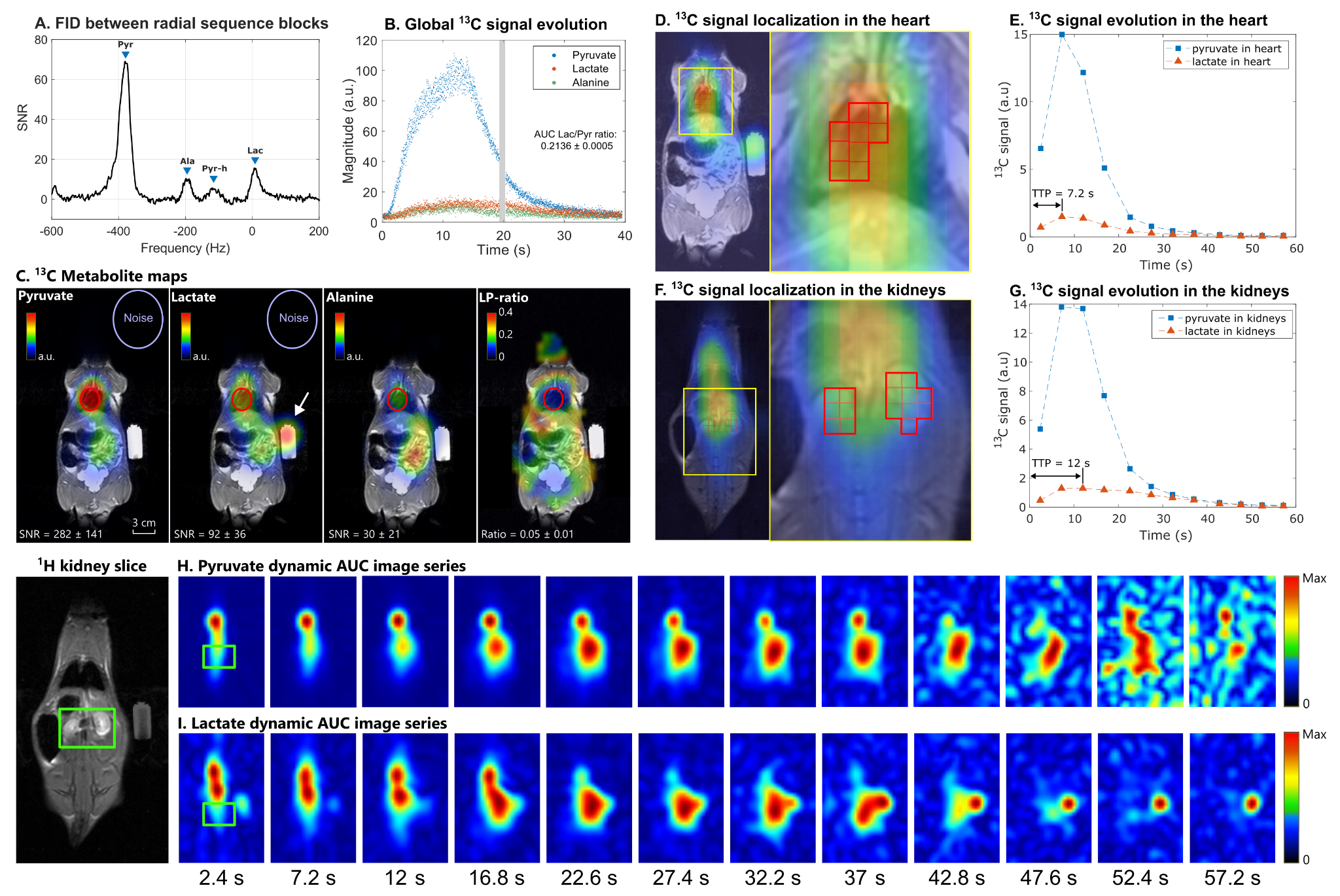


**Figure S7.** Results for rat M3


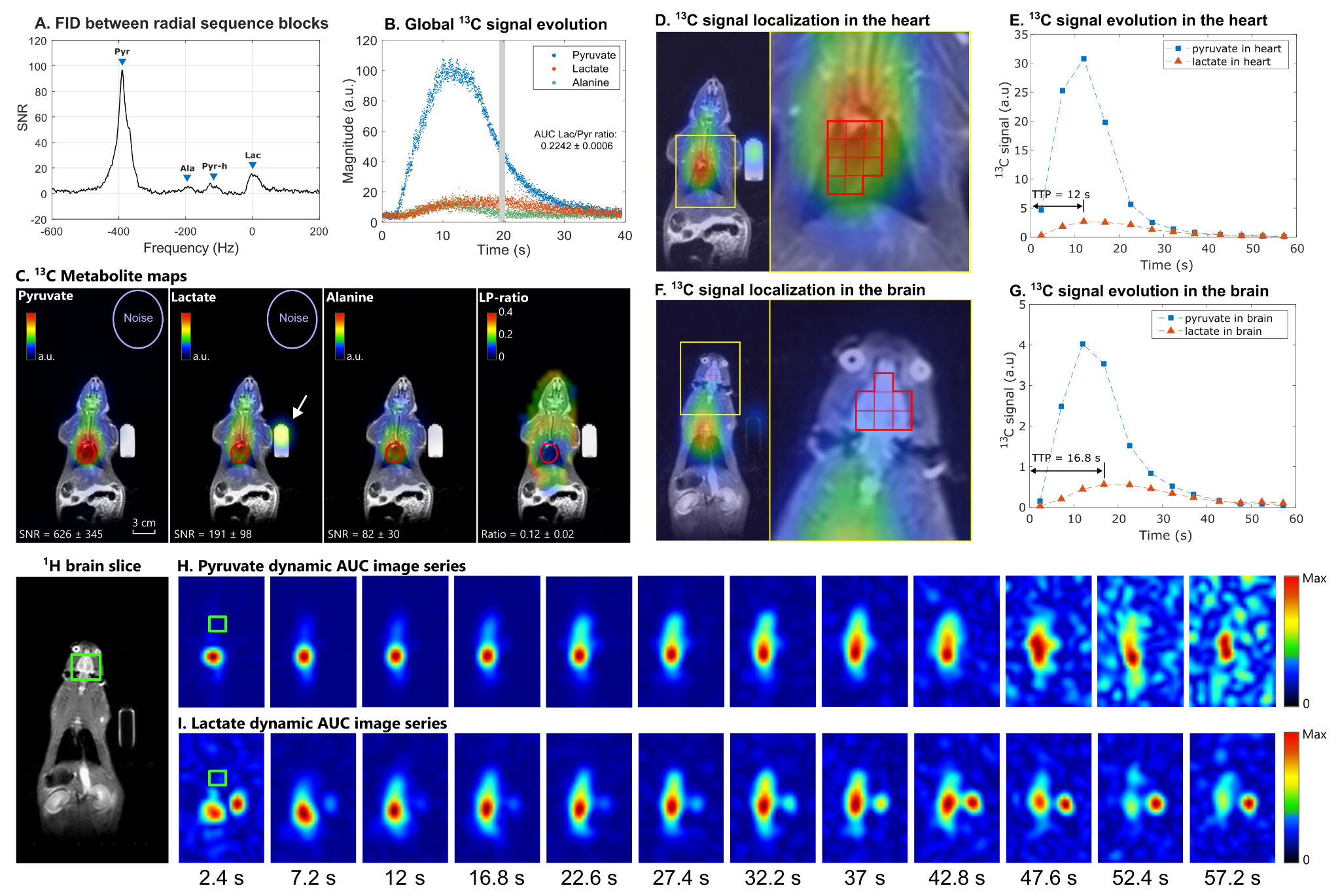


###

### **3.1. 3D Cartesian ME-bSSFP Sequence & Post-processing**

Using another female healthy rat (F2) injected with HP pyruvate, the pyruvate-to-lactate metabolism was again measured dynamically with a 3D Cartesian phase-encoded ME-bSSFP sequence and IDEAL in image space. Various factors, including the fact that different subjects have been used, prevent a direct comparison to the radial ME-bSSFP. However, it is still possible, to some extent, to validate the sequence and data processing methods we previously implemented and to observe some advantages of radial sampling over the conventional Cartesian approach.

The 3D Cartesian ME-bSSFP (**Figure S8A**) was designed with refocusing excitation flip angles ±α (after an initial α/2 preparation), five bipolar gradient echoes centered around TR/2 between RF pulses. The multi-echo raw signals from Cartesian ME-bSSFP went through a different post-processing pipeline than the radial data (**Figure S8B**).


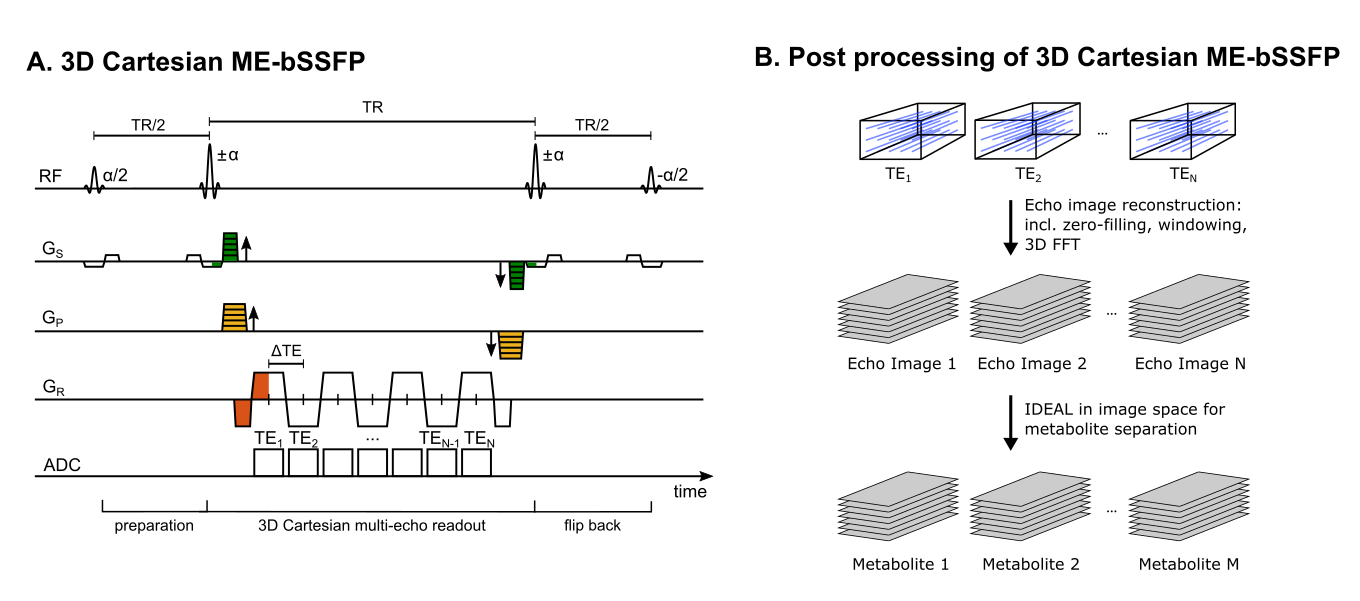
**Figure S8. A.** Schematic representation of the dynamic 3D Cartesian ME-bSSFP with bipolar multi-echo readout in one direction and phase-encoding in orthogonal and slice direction. **B.** The post-processing routine of the Cartesian ME-bSSFP included echo image reconstruction and IDEAL decomposition in image space to obtain the individual metabolite maps.

### **3.2. *In vitro* experiment: Radial vs. Cartesian**

Thermally polarized *in vitro* MRI experiments with the ^13^C-labeled reference solutions were conducted on a 3T Prisma (Siemens Healthineers) equipped with a dual-tuned ^1^H/^13^C-transmit/receive volume coil (Rapid Biomedical). The used solutions, [^13^C]urea and [1-^13^C]lactate, are identical to the ones mentioned in the main text. The MRI parameters were set as close as possible for the radial and Cartesian versions of ME-bSSFP for comparison: alternating flip angles *α* = ±60° after an initial *α*/2-TR/2 preparation block, repetition time TR = 16 ms, readout bandwidth 1000 Hz/px, FOV = 300 × 150 × 94 mm, base resolution: base resolution of 32 (oversampling factor of 2) for radial, resolution of 32 × 16 × 10 for Cartesian.

Applying the IDEAL algorithm, the ^13^C signals from the thermally polarized *in vitro* phantoms (**Figure S9A** showing ^1^H MRI) measured with both ME-bSSFP were successfully decomposed into those from urea and lactate at a frequency shift of 0 Hz and +608 Hz, respectively. The reconstructed ^13^C maps show a reliable separation of the metabolite signal contributions, which is indicated by the localization of the signals of the two high-concentration metabolite solutions (radial: **Figure S9C, S9D;** Cartesian: **Figure S9E, S9F**). The frequencies for the decomposition were obtained from a non-localized ^13^C spectrum measured priorly (**Figure S9B**).

**
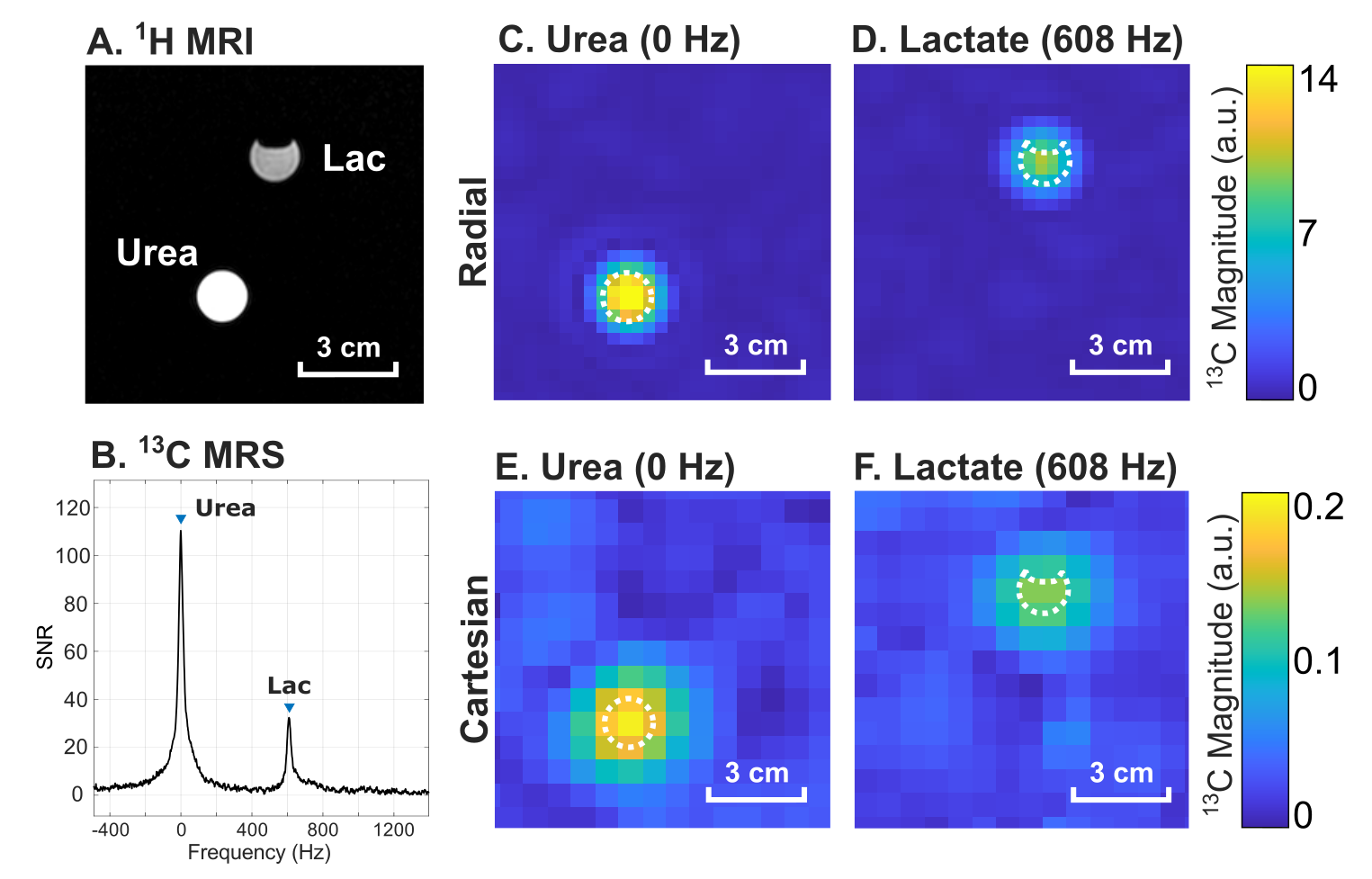
**

**Figure S9.** Thermally polarized *in vitro* measurements with radial and Cartesian ME-bSSFP were successfully reconstructed using IDEAL at the corresponding frequencies of 0 Hz (**C**, urea, SNR: 18) and +608 Hz (**D**, lactate, SNR: 13), yielding the separated metabolite maps.

### **3.3. *In vivo* experiment (Rat F2, Cartesian)**

For *in vivo* hyperpolarized ^13^C acquisition, the healthy female Wistar rat (F2) (m = 286.9 g, Charles River, 17 weeks old) was injected with a volume of 5.5 mL/kg BW via the tail vein catheter for the Cartesian scan. The peak resonance frequencies, [1-^13^C]pyruvate (220 Hz), [1-^13^C]lactate (590 Hz), were accurately obtained from FID spectroscopy acquisitions that were interleaved between the ME-bSSFP scans. The ^13^C MRI parameters were set referring the radial sequence session: alternating flip angles *α* = ±10°, repetition time TR = 16 ms, readout bandwidth 1000 Hz/px, FOV = 356 × 178 × 62.5 mm, base resolution: matrix size of 32 × 16 × 10.

As shown in **Figure S8B**, the complex echo images were first reconstructed. The matrix size was extended by zero-filling to 64 × 32 × 10, resulting in a spatial resolution of 5.56 × 5.56 × 6.25 mm³. This was followed by the separation of metabolite intensities via IDEAL in image space. The metabolite frequencies used for this experiment were 0 Hz for urea, +220 Hz for pyruvate, and +590 Hz for lactate. *In vivo* ^13^C spectroscopy (**Figure S8A**) was performed at 25.6 s after the sequence start, revealing the peak resonance frequencies of [1-^13^C]pyruvate (220 Hz) and [1-^13^C]lactate (590 Hz). The [^13^C]urea (0 Hz) peak was used for central frequency calibration with the aid of the thermally polarized [^13^C]urea solution before the start of the experiment.

### ***In vivo* results (Rat F2, Cartesian)**

After IDEAL decomposition of the metabolite signals, the global metabolite intensities were calculated by spatially summing the signals over the whole FOV (**Figure S10B**). This allowed us to dynamically monitor the global (non-localized) metabolic conversion. The Cartesian sampling provides a temporal resolution of 2.56 s, depicting the injection bolus and conversion of pyruvate into lactate.

The ^13^C metabolite maps and ^1^H anatomical MRI are displayed in **Figure S10** (central coronal slice of heart) and **Video S2** (full slices). The accumulation of HP pyruvate signals and subsequent lactate formation is again observable in the head, heart, and abdomen. The ^13^C-labeled urea reference phantom is highlighted. Voxel-wise division of the summed 3D metabolite maps yielded the AUC ratio map. The map identified areas of high conversion from pyruvate to lactate, e.g., heart and head.

**
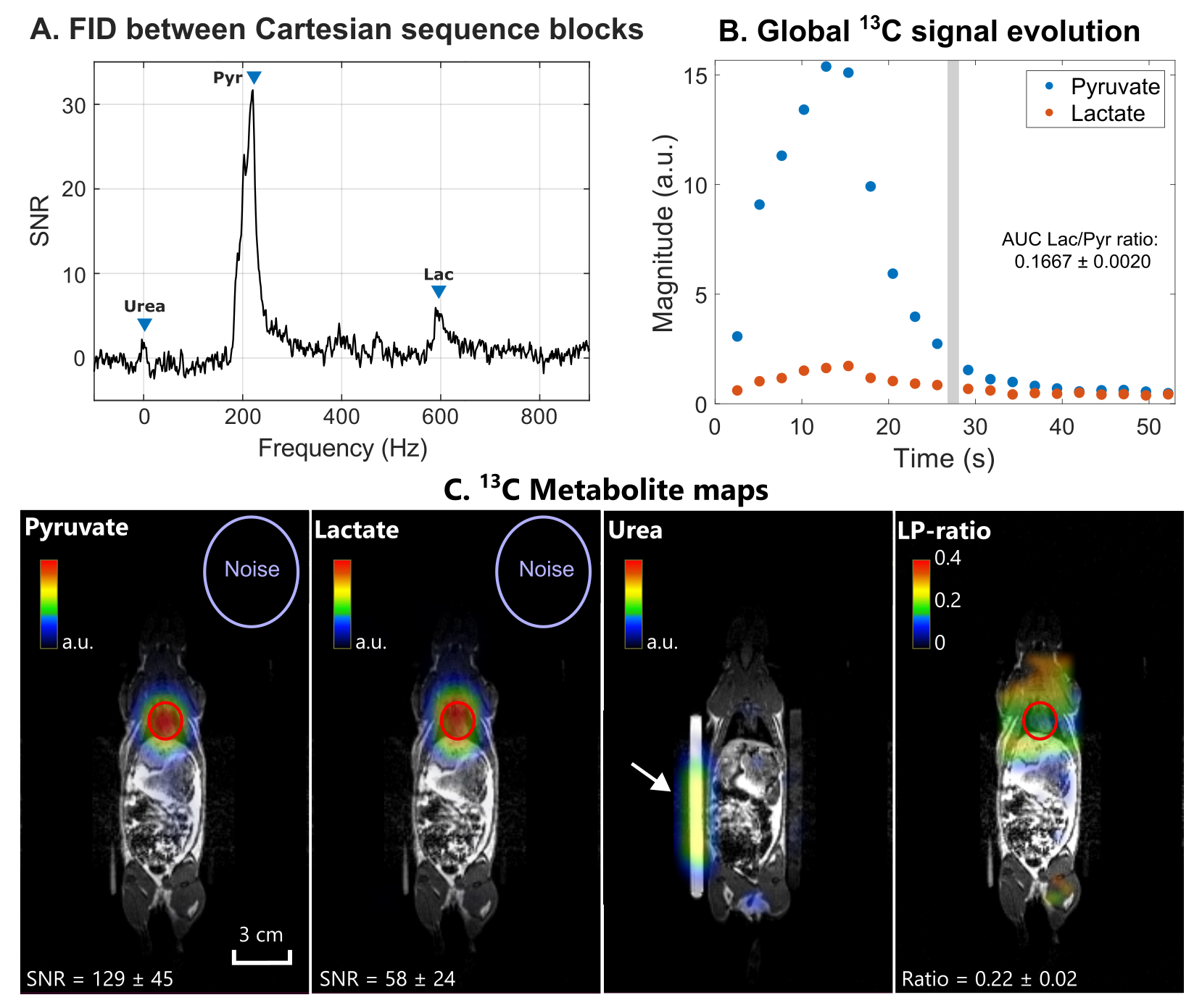
**

**Figure S10. A.** *In vivo* ^13^C spectroscopy at 25.6 s after the sequence start, revealing the peak resonance frequencies of [1-^13^C]pyruvate (220 Hz) and [1-^13^C]lactate (590 Hz). The thermal [^13^C]urea (0 Hz) peak is fixed at 0 Hz as prior knowledge from the thermally polarized [^13^C]urea solution. **B.** Global signal level with time in every decomposed image. The Cartesian sampling provides a temporal resolution of 2.56 s, roughly depicting the injection bolus and conversion of pyruvate into lactate. **C.** Central coronal slice of heart from HP ^13^C metabolite AUC images, obtained from Cartesian ME-bSSFP acquisition after injection of HP pyruvate into the rat F2, showing metabolic conversion from pyruvate to lactate. A slice at the position of the reference solution highlights the thermally polarized [^13^C]urea next to the animal. Voxel-wise division of metabolite maps and applying a mask based on the pyruvate signal yielded the lactate-to-pyruvate ratio map.

**Videos (separated files)**:

Videos are provided as supplementary materials for better visualization of the three-dimensional images. The repository contains:

- **Video S1**: Folder “***Radial 3D 13C Images***”: cumulative 3D metabolite images (pyruvate, lactate, alanine) and 3D lactate-to-pyruvate, alanine-to-pyruvate metabolite ratio map from radial ME-bSSFP acquisitions of four rats (F1, M1, M2, M3).
- **Video S2**: Folder “***Cartesian 3D 13C Images***”: cumulative 3D metabolite images (pyruvate, lactate) and 3D lactate-to-pyruvate metabolite ratio map from Cartesian ME-bSSFP acquisition of rat F2.
